# Pre-stroke dementia and in-hospital outcomes in the Chinese Stroke Center Alliance

**DOI:** 10.1093/geroni/igaa057.3250

**Published:** 2020-12-16

**Authors:** Chelsea Liu, Hong-Qiu Gu, Xin Yang, Chun-Juan Wang, Kai-Xuan Yang, Zi-Xiao Li, Yong-Jun Wang

**Affiliations:** 1 Harvard School of Public Health, Boston, Massachusetts, United States; 2 Beijing Tiantan Hospital, Beijing, China

## Abstract

Little is known about the prevalence of pre-stroke dementia in China and whether this group is at higher risk of adverse in-hospital outcomes. We aimed to understand this association using data from the Chinese Stroke Center Alliance. Multivariable logistic regressions were conducted to assess the association between pre-stroke dementia status and ambulation at day 2, in-hospital mortality, and in-hospital complications. Covariates included age, sex, medical history, history of smoking, history of alcohol use, medication history (antiplatelet drugs, lipid-lowering drugs), stroke severity (measured by the National Institute of Health Stroke Scale), whether IV tPA was administered within 4.5 hours, and whether the patient received deep vein thrombosis prophylaxis as needed. Odds ratios and 95% confidence intervals were presented for the adjusted models. In the final analytic sample of 559,070 ischemic stroke patients with no prior stroke history enrolled across 1476 hospitals, 1511 (0.3%) had pre-stroke dementia, and they were older and more likely to be female. Patients with pre-stroke dementia had lower odds of ambulating at day 2, higher odds of having any complications and higher odds of in-hospital mortality compared to those without pre-stroke dementia, despite little difference in treatment received. Our findings may be explained by communication barriers experienced by patients with pre-stroke dementia that limited their ability to advocate for their own care needs. Further research is needed to determine whether a different care pathway or additional attention from clinicians is necessary for patients with pre-stroke dementia.

